# STAMP (Supporting Treatment and Appropriate Medication in Paediatrics) to STOMP (Stopping Over Medication of People With a Learning Disability and Autistic People) – A Review of the Demographic and Clinical Characteristics of Transitions From CAMHS to Adult MHLD and Their Outcomes

**DOI:** 10.1192/bjo.2025.10513

**Published:** 2025-06-20

**Authors:** Sajitha Nair, Mogbeyiteren Eyeoyibo

**Affiliations:** 1 Kent and Medway NHS and Social Care Partnership Trust, Ashford, United Kingdom; 2 Kent and Medway NHS and Social Care Partnership Trust, Dartford, United Kingdom

## Abstract

**Aims:** This study investigates the demographic and clinical characteristics of young individuals (aged 17–24) transitioning from CAMHS (Child and Adolescent Mental Health Service) to Adult MHLD (Mental Health of Learning Disability) services in North Kent. It examines the reasons for psychotropic medication use, assesses medication burden, and evaluates MHLD’s effectiveness in reducing or discontinuing unnecessary prescriptions.

**Methods:** A retrospective review was conducted by searching the records of patients registered with MHLD North Kent between 2011 and 2022. The study included individuals aged between 17–24 years at their first MHLD assessment, either referred from CAMHS or via GP, Community Learning Disability Team, or Community Mental Health Team. Those first seen after age 24 were excluded. Data analysis covered referral sources, demographics, co-morbidities, prescribing patterns, and treatment outcomes.

**Results:** Seventy-one patients were identified, with an average referral age of 19. Males comprised 65%. 82% were White British. Learning disabilities were classified as mild (38%), moderate (39%) or severe (23%), with 87% having autism and 32% diagnosed with ADHD. Epilepsy was noted in 25%. Psychotropics were primarily prescribed for behavioural challenges, with risperidone being most common (32%), followed by promethazine (30%), melatonin (23%), and aripiprazole (15%). Medication reduction was attempted in 27% of cases, with 18% achieving successful dose reduction or discontinuation. Psychological interventions were provided to 55% of patients, with 36% having a diagnosis of challenging behaviour. Importantly, no patient exceeded a psychotropic load of 100%.

**Conclusion:** The main reason for referral was challenging behaviour. Psychotropic prescribing was frequent, yet no direct link was found between prescribing patterns and demographic factors. The MHLD team successfully maintained psychotropic loads within safe limits and engaged over half of the patients in psychological therapies. While medication reduction efforts were undertaken, success rates remained modest.

Recommendations:

Strengthen medication monitoring systems to enhance reduction efforts.

Develop a structured STOMP/STAMP plan and share it with GPs and carers.

Regularly review care plans, particularly when side effects arise.

Improve access to MHLD services for GPs and carers to build confidence in medication management.

Work closely with psychological services to address challenging behaviour at its source.

Implement a clear medication review flowchart, incorporating it into patient records and communication with primary care.

These steps aim to enhance care for individuals with intellectual disabilities and refine medication management within MHLD services.

